# Synthesis of findings from the literature and a qualitative research study on the impacts of gender, disability, and ethnicity in Neglected Tropical Diseases programs

**DOI:** 10.1371/journal.pntd.0011782

**Published:** 2023-12-04

**Authors:** Jennifer K. Arney, Maureen K. Headland, Andrea M. Bertone, Aboulaye Meite, Virginie Ettiegne-Traore, Kofi Asemanyi-Mensah, Irene Dede Teiko Dzathor, Ibrahim Kargbo-Labour, Umu Jalloh, Patricia Houck, Diana Stukel

**Affiliations:** 1 FHI 360, Washington, DC, United States of America; 2 Programme National de Lutte Contre les Maladies Tropicales Négligées à Chimiothérapie Préventive, Ministry of Health, Abidjan, Côte d’Ivoire; 3 FHI 360, Abidjan, Côte d’Ivoire; 4 Neglected Tropical Diseases Programme, Disease Control and Prevention Department, Ghana Health Service, Public Health Division, Accra, Ghana; 5 FHI 360, Accra, Ghana; 6 Neglected Tropical Disease Programme, Ministry of Health and Sanitation, Freetown, Sierra Leone; 7 Helen Keller International, Freetown, Sierra Leone; 8 Helen Keller International, NYC, New York, United States of America; Kwame Nkrumah University of Science and Technology, GHANA

## Abstract

**Introduction:**

Act to End NTDs | West, a USAID-funded program that supports national governments to eliminate or control five neglected tropical diseases (NTDs) in West Africa including trachoma, lymphatic filariasis (LF), onchocerciasis, schistosomiasis and soil-transmitted helminthiasis, conducted a gender and social inclusion analysis to determine how NTDs differentially impact various populations and how gender and social norms impact NTD programs to inform future programming.

**Methods:**

The study used a mixed methods approach including a literature review; primary qualitative data collection; and monitoring data in Côte d’Ivoire, Sierra Leone, and Ghana.

**Results:**

Women and girls face additional health risks from many NTDs compared to men and boys. In addition to differential health burden, the social and economic impacts of NTD-related disability or infertility can be particularly dire for women and girls.

Men were somewhat less likely to participate in mass drug administration (MDAs) due to: lack of information about campaigns, lack of access due to work, and higher levels of mistrust of the government and concerns about side effects of the medicines. Pregnant and breastfeeding women were sometimes excluded by community drug distributors (CDDs) from certain types of MDAs for which they are eligible.

Training participation rates for CDDs and supervisors were nearly universally higher for men than women, even though feedback on the effectiveness of female CDDs was overwhelmingly positive, and female CDDs often have more access to other women in conservative households. The role of a CDD can lead to career and social opportunities for women. However, challenges faced by CDDs were seen as a greater barrier for women, including transportation, safety, household responsibilities, lower education levels, and low or lack of wages.

**Discussion:**

Programs to address NTDs can promote equity and improve programming by increasing women’s participation as CDDs and providing financial compensation. Additionally, programs should prioritize inclusive training for CDDs, and inclusive messaging about MDA for communities.

## Author summary

The Act to End NTDs | West program supports USAID’s efforts to control or eliminate trachoma, lymphatic filariasis (LF), onchocerciasis, schistosomiasis and soil-transmitted helminthiasis in 11 West African countries using mass drug administration.Act | West conducted a gender and social inclusion analysis to inform a strategy for the program to improve outcomes and more meaningfully engage and empower women and marginalized individuals in program implementation. Our objective was to identify how gender and social norms and stigma differentially impact men’s and women’s adoption of healthy practices around NTD prevention, use of NTD services, participation in programing, and opportunities to engage in identifying solutions, using an intersectional lens inclusive of disability, ethnic identity, and geographic location.

Several studies have looked at gender dimensions of staffing for NTD programming, analyzed differential risk factors and prevalence of NTDs between males and females, or presented data on gender and national mass drug administration (MDA) coverage to combat NTDs. This paper aims to consolidate these findings through a literature review as well as a qualitative study conducted in Côte d’Ivoire, Ghana, and Sierra Leone, to provide a comprehensive look at gender and social inclusion within NTD programming across various contexts and diseases.

This study found that women and girls face additional health risks from five NTDs compared to men and boys. In addition to the health burden, the social and economic impacts of NTD-related disability or infertility can be particularly severe for women and girls.

Additionally, men were somewhat less likely to participate in mass drug administration (MDAs) and pregnant and breastfeeding women were sometimes excluded by community drug distributors (CDDs) from certain types of MDAs for which they are eligible due to misunderstanding on the part of CDDs and community members on eligibility criteria.

Training participation rates for CDDs and supervisors were universally higher for men than women, even though feedback on the effectiveness of female CDDs was overwhelmingly positive, and female CDDs often have more access to other women in conservative households. Additionally, challenges faced by CDDs were seen as a greater barrier for women to serve in that capacity.

## Introduction

The United States Agency for International Development’s (USAID) Act to End Neglected Tropical Diseases (NTDs) | West (Act | West) program is a five-year USAID-funded cooperative agreement that seeks to eliminate or control five NTDs (lymphatic filariasis [1], trachoma [2], onchocerciasis [3], schistosomiasis [4], soil-transmitted helminths [5]) in 11 West African countries: Benin, Burkina Faso, Cameroon, Ghana, Guinea, Ivory Coast, Mali, Niger, Senegal, Sierra Leona and Togo. The program supports national governments to roll out mass drug administration (MDA) campaigns to treat all eligible individuals in an affected community with drugs that both treat the disease in those who are infected, as well as protect those who aren’t from future infection. These campaigns are primarily carried out by community drug distributors (CDDs) who are trained by government health teams to raise awareness of NTDs and the drugs used to treat them, as well as ensure all eligible individuals participate in the MDA campaigns.

As a way to ensure the program is equitably addressing the needs of men, women, boys and girls with NTD control and elimination activities, Act | West conducted a gender and social inclusion (GESI) analysis study in 2019 to determine how NTDs differentially impact various populations and how gender and social norms and power differentials between men and women might impact results, with a view to informing future NTD programming, integrating elements to explicitly advance gender equality and social inclusion. The GESI analysis took an intersectional approach, looking not just at how gender norms and roles impact various components of NTD programming, but also looking at ethnicity, geographic context, urban vs. rural, and disability.

We have consolidated several original research studies and metanalyses that have looked at gender dimensions of human resources for NTD programming; studies that have analyzed differential risk factors and prevalence of NTDs between males and females; and sex-disaggregated national level MDA coverage data. This paper aims to provide a more comprehensive look at gender and social inclusion within NTD programming across various contexts, diseases, and implementation of MDA.

## Methodology

### Ethics statement

Prior to fieldwork, research team members underwent training on best practices in human subject research ethics, gender analysis data collection, data entry and cleaning, and qualitative analysis prior to data collection. All individuals who participated were provided informed consent prior to the start of the interview, and written consent was obtained from all participants who were able to sign their name. Verbal consent was obtained for any participants who were not able to sign their name. The protocol for this study, data collection instruments, and consent forms were approved by FHI 360’s Protection of Human Subjects Committee and local research ethics boards in each of the three study countries (Comite National d’Ethique des Sciences de la Vie et de la Sante in Côte d’Ivoire; Ghana Health Service Ethics Review Committee on Research Involving Human Subjects in Ghana; and the Office of the Sierra Leone Ethics and Scientific Review Committee in Sierra Leone).

The objectives of the gender and social inclusion analysis were to identify the following:

- How NTDs might differentially impact women, men, and school-aged children 6–15 years old, recognizing intersectionality such as disability, ethnicity, etc.;
- How gender norms, roles, and power dynamics, including social exclusion of people with disabilities, might affect the attainment of program results; and
- How program activities might advance gender equality and social inclusion and promote sustainable health outcomes in the context of NTD control and elimination programming.

The gender and social inclusion analysis used a mixed methods approach, including a literature review of secondary data and primary data collection using qualitative methods. The team also drew on the quantitative analysis documented in *Gender equity in mass drug administration for neglected tropical diseases*: *data from 16 countries* [6], as well as other programmatic monitoring data. Qualitative data was gathered through key informant interviews (KIIs) and focus group discussions (FGDs) in program sites in Côte d’Ivoire, Sierra Leone, and Ghana from May to July 2019.

The literature review consisted of an internet-based search of published and grey literature on gender and NTDs, current and previous program reports, and technical documents produced by donors and implementing organizations. Relevant peer-reviewed literature was identified through searching three electronic databases—PubMed, Embase, and Cumulative Index to Nursing and Allied Health Literature (CINAHL). Search terms included gender, women, men, girls, boys, ethnicity, religion, social inclusion, disability, CDD, MDA, NTD, trachoma, schistosomiasis, soil-transmitted helminths, lymphatic filariasis, and West Africa. We also conducted secondary reference searching of review articles identified in the search for additional relevant publications.

For country-level data collection, we purposively selected the three countries (Côte d’Ivoire, Sierra Leone, and Ghana) to be as representative as possible of the 11 West African program countries, including demographic data such as religious and ethnic make-up. We also selected countries based on percentage of women trained as CDDs, types of MDA present, length of NTD program implementation, and security considerations.

The key informant interviews and focus group discussions totaled 477 individuals across the three study countries. Seventeen KIIs were conducted across the three countries, including with in-country program staff, government officials involved in NTD programming, members of international organizations involved in NTD programming, including disabled persons groups, and members of local community-based or civil society organizations involved in NTD programming.

Twenty-one FGDs were conducted in each country. Each FGD consisted of 6−8 participants from each of the following groups:

- 3 groups of community leaders (mixed male and female)
- 6 groups of community drug distributors (CDDs) (3 females and 3 males in each country)
- 3 groups of health providers (mixed male and female)
- 3 groups of mothers of school aged children (6–15 years old)
- 3 groups of fathers of school aged children (6–15 years old)
- 3 groups of grandmothers of school-age children (6–15 years old)

These participant groups were selected based on their role in decision-making and participation in both community-based and school-based MDA campaigns.

Data was transcribed into English from audio recordings, and triangulated using notes, into Microsoft Word. Microsoft Excel was used for efficient analysis, and then coded according to the domains of gender analysis from USAID’s *ADS Chapter 205*: *Integrating Gender Equality and Female Empowerment in USAID’s Program Cycle* [7] adapted to the context of NTD programming. FGDs were analyzed collectively by population group, and KIIs were analyzed collectively as one group.

A codebook was developed by the Principal Investigator and co-investigators in an iterative process. The study team used the process of circular coding including information collected from literature and raw data, using grounded theory. No double-coding was done.

Data was coded and analyzed according to the domains of gender analysis. The following domains were applied: policies and regulations; patterns of power and decision making; gender-related health and social impacts of NTDs; access to information about NTDs and MDAs; access to and acceptability of MDAs; and cultural norms and beliefs impacting the selection and roles of male and female CDDs. Results presented below are findings from both the global literature review as well as the qualitative findings from the primary data collection in Sierra Leone, Ghana, and Côte d’Ivoire.

### Study sites

The research study took place in the capital cities and one or more communities in nine selected districts: three each in Côte d’Ivoire, Sierra Leone, and Ghana. These districts were chosen based on overall program coverage, sex-based differences in program coverage, representativeness across NTDs, presence of lymphatic filariasis (LF) hotspots (an area that is smaller than an evaluation unit that has an Ab (antibody) prevalence (upper 95% confidence limit) of >2% despite five or more rounds of effective MDA), religious ethnic group distribution, urban and rural dynamics, type of local economies, and border areas/migrant populations. In Côte d’Ivoire, the sites selected were Abidjan and communities in Korhogo, Divo, and Touba districts. In Ghana, the sites selected were Accra and communities in Kpandai (Northern Region), Sekyere Afram Plains (Ashanti Region), and Ahanta West (Western Region). In Sierra Leone, the sites selected were Freetown and communities in Koinadugu, Kenema, and Western Area Rural (WAR) districts. Table 1 below provides a breakdown of the selected districts in each country.

**Table 1:** District profiles for study sites.

Regions	Districts	Primary economy	Migrant/ nomadic populations	MDA types*	Rural vs. urban	Major ethnic groups
**Côte d’Ivoire**						
Kabadougou-Bafing-Folon	Touba	Agriculture, factory	No	LF; OV; STH	Rural	Malinke
Lôh-Djiboua	Divo	Agriculture, factory, commerce	Yes	LF; OV	Peri-urban	Dida, Abe, Baoule
Poro-Tchologo-Bagoue	Korhogo	Mining, agriculture	Yes	LF; OV	Rural	Senoufo
**Sierra Leone**						
Northern	Koinadugu	Agriculture	Yes	LF; OV; STH	Rural	Kuranko, Mandinka, Fulani, Limba, and Yalunka
Eastern	Kenema	Gold mining, agriculture, commerce	Yes	LF; OV; STH	Peri-urban	Mende
South	Western Area Rural (WAR)	Fishing, agriculture, commerce	Yes	LF; STH	Urban	Krio, Sherbro
**Ghana**						
Western	Ahanta West	Agriculture	No	LF hotspot; OV; SCH	Rural	Akan
Northern	Kpandai	Agriculture	No	OV	Rural	Gur, Guan
Ashanti	Sekyere Afram Plains	Agriculture	No	OV; STH; SCH	Rural	Akan, Fulani

*MDA = mass drug administration; LF = lymphatic filariasis; OV = onchocerciasis; STH = soil transmitted helminths; SCH = schistosomiasis

## Results

The results of this gender analysis are presented as a combination of findings from the literature review, program monitoring data, and the primary qualitative data collection. Within each following subsection of the results, literature review and monitoring data are presented first to provide context to the qualitative study findings.

### Vulnerability to NTDs related to gender roles

Other than trachoma, the rates of infection for NTDs are generally comparable across sexes [8]. An estimated 157.7 million people living in trachoma-endemic areas are at risk [2]. Trachoma infection rates are most disproportionate by sex, compared with other NTDs [9], with girls and women being two to four times more likely to be infected and twice as likely as men and boys to develop trichiasis [10]. Gender role factors, rather than biology likely explain these differences in infection such as caretaking of infected children [11].

Unlike trachoma, the risk of developing schistosomiasis (SCH) [4] is relatively more equal between genders though the sources of the risk are different based on proscribed roles. Activities such as washing clothes and fetching water may expose women and girls to increased risks of developing SCH in endemic areas [6,12,13]. This is particularly true since two-thirds of water collection is performed by women and girls [14]. However, in some instances men and boys may be at increased risk due to occupational exposure. Men and boys are most often at risk when carrying out their productive roles. This is true for SCH where occupational activities such as fishing and farming put men at increased risk of contracting the disease [12,15,16]. In Ghanaian communities along irrigation canals, male school children had a higher infection level of SCH than girls. While both boys and girls were exposed to the water, significantly more males than females swam in the canals, washed their clothes there, and worked on rice farms. More females reported washing dishes in the canals, but this activity held less exposure to infection [17]. Another study found an increased risk for LF among males who hunt or fish, particularly at night [18].

### Differential physical impacts of NTDs on women and men

However, while for most NTDs males and females may experience similar infection rates, women and girls face additional risks of negative health impacts as a result of NTDs. These include increased anemia during pregnancy brought on by soil-transmitted helminths (STH) infection [5,19].

Schistosomiasis affects both maternal and infant morbidity and mortality. In Africa, an estimated 10 million pregnant women are currently infected with SCH, and half of those will go on to develop severe anemia and associated complications, including low birth weight infants and increased maternal and infant mortality [20]. Schistosomiasis can be transmitted via the placenta, resulting in congenital infection of newborns. Pregnant women infected with SCH may also experience higher rates of spontaneous abortions and ectopic pregnancies [21].

Women infected with SCH or hookworms (one of three STHs, the other two being roundworms and whipworms) are more vulnerable to severe anemia [15,22]. Hookworm-related anemia is a form of iron deficiency anemia caused by gastrointestinal blood loss as a result of the feeding activity of intestinal hookworms [23].

In addition to anemia and poor maternal and infant outcomes, both hookworms and SCH contribute to infertility. Hookworm-related anemia causes amenorrhea, and genitourinary SCH leads to inflammation of the uterus, fallopian tubes, and ovaries [24]. Up to 3.6% of ectopic pregnancies and 41% of infertility cases are attributed to female genital SCH in endemic areas [25].

Lymphatic filariasis is the second leading cause of permanent disability worldwide [26]. Women with the disease are particularly prone to lymphedema, while men are susceptible to hydrocele.

### Differential social and economic impacts of NTDs by sex

The social consequences of NTD infections, including stigma, impact men and women differently and may disproportionately affect women, particularly when NTD infections negatively affect socially ascribed assets and attributes for femininity such as beauty, marriage, motherhood, and childcare.

### Fertility-related social impacts

As described in the section above on physical impacts of NTDs, schistosomiasis can often lead to female infertility. Infertile women may suffer discrimination, stigma, and ostracism from their partners, families, and the broader community because of a perceived inability to continue the family line or contribute to the economic well-being of the community [27]. In some settings, women who do not demonstrate fertility may be divorced, set aside for second wives, or suffer verbal and physical abuse. Infertility has adverse psychosocial and economic implications for affected women/families in most developing countries [27].

### Caretaking responsibilities

Across West Africa, it is the socially expected role of women and girls to take care of ill or disabled family members who need regular assistance [28]. This was confirmed during the project’s qualitative data collection where, almost universally, study participants confirmed that women and girls are responsible for caring for younger children and other family members in their household who are not able to care for themselves. In addition to the potential negative consequences of NTD-related illness and disability for oneself if infected, women and girls carry an extra burden if a family member is ill or disabled by an NTD.

### Stigma and discrimination

One systematic review on NTDs and social stigma found that being female, young, having genitals affected, being poor or having advanced infection all are associated with higher levels of stigma [29]. This same review found fourteen studies that cited inability to fulfill a certain gender role as leading to stigmatization, where that inability included reproducing, having sexual relations, and performing household chores. Hofstraat et al. also noted that while higher levels of stigma were associated with being female, men also experience stigma due to LF-associated hydrocele and the inability to fulfill gender roles. Other studies have found that women with LF-associated lymphedema, disfigurement from onchocerca skin disease (one of the morbidities associated with onchocerciasis [3]) and trachoma-associated trichiasis experience social ostracization resulting in fewer opportunities for marriage [15,24,30–32].

According to the literature, trichiasis (caused by trachoma and manifested in vision impairment or complete blindness and particularly affecting women) has a significant negative impact on affected women’s quality of life, including experiencing stigma and discrimination [29]. Trichiasis affects women’s ability to marry, enjoy a social life, have good relationships, be employed, or participate in religious obligations [32]. Affected women experience self-stigma through internalized feelings of shame while perpetrators of external stigma justify their behavior by attributing it to the affected person’s perceived contagiousness, inability to fulfill gender roles, and being a social and financial burden to the family. For these reasons, women experience a precipitous decline in their independence [32]. More recent studies have found similar impacts of onchocerciasis and trachoma blindness or vision impairment on women’s employment, mobility, and social lives [13].

Lymphedema, which is caused by LF and manifests as extreme swelling of the limbs or other parts of the body, occurs more frequently in women than in men and often involves the breasts and genitals [24]. Within the GESI analysis, both male and female community FGDs in Ghana described situations where men had abandoned their wives because the wives had visible symptoms of lymphedema. However, none of the women who participated in the FGDs mentioned that they knew of any woman who left her husband because he had symptoms of an NTD. A few study respondents stated that women with lymphedema would hide their disability by staying in the house (and over time become socially excluded), but that men with hydrocele continued to be out in the community.

Our research found that some of the stigma related to NTDs is due to community misunderstandings around how NTDs are acquired. For instance, FGDs with male community leaders in Kpandai district, Ghana, revealed their belief that LF was passed directly from person-to-person and that people contract the disease by being close to the infected person—which undoubtedly increases stigma towards people with LF-related disabilities.

### Economic impacts

In addition to limiting marriage opportunities, stigma and discrimination related to disability and disfigurement resulting from NTD infection limits women and girls’ employment opportunities, further impacting their economic wellbeing and independence [24]. Among FGD participants in Ghana, mothers and community leaders reported that some women with lymphedema worked in the markets as food sellers, but no one would buy food from them, and they eventually lost their livelihoods.

### Gender differences in community-based MDA coverage rates

According to available quantitative data across various countries implementing MDA programming for NTDs, program coverage between males and females has been largely equal at the national level, but the pattern is less clear at sub-national levels [6]. In our review of the literature and program monitoring data, we found mixed results depending on disease, country, and year. A few studies suggested that men may be less likely to participate in MDAs, in part due to conflicting priorities when work or occupational travel draws them away from MDA sites [6], but the findings were not necessarily generalizable across different countries and sites.

Women who are pregnant are not currently eligible to participate in ivermectin-based MDA (for onchocerciasis and LF) due to safety concerns, although this guidance may be updated in the future due to clinical studies not finding higher pregnancy-related risks from doses of ivermectin used in MDA [33]. For women who have multiple pregnancies close together, they often miss out on MDAs for multiple years in a row. Several women across the three study regions in Ghana reported that they had never taken ivermectin because they had been pregnant or breastfeeding every time an MDA occurred. This is concerning given that it doesn’t match the official WHO and government guidelines that state that while it is not recommended for pregnant women to take ivermectin for onchocerciasis prevention, it is safe for breastfeeding women more than one week post-partum to ingest ivermectin. WHO encourages inclusion of pregnant and lactating women in SCH and STH MDA, which do not use ivermectin, yet in practice many national programs still do not target them for treatment because of perceived risks. Lack of knowledge on correct guidance and effective messaging among CDDs regarding safety of MDA for pregnant and lactating women can result in the exclusion of women eligible for treatment [15,34].

According to the 2016 district-level data analysis from 16 countries [6], the median MDA coverage for all targeted diseases was slightly higher for females than males when aggregated across all countries (ranging from 9.4% higher for LF MDA to 2.3% higher for trachoma MDA). When analyzed by country, district-level MDA coverage was higher among females in all countries except Haiti and Mozambique. Coverage among females was more than 10% higher in Nigeria, and more than 5% higher in Burkina Faso, Senegal, Niger, and Tanzania [6].

### Access to MDAs

MDAs in districts that border neighboring countries tend to miss community members who travel between countries during distributions. These may include both men and women (with men traveling alone more for commerce/work, and women and families traveling for life events such as funerals, weddings, etc. or entire families traveling in the case of nomadic communities). However, both the qualitative study and program monitoring data showed that MDA coverage is lower for males than females in border districts. In Kenema and Koinadugu districts in Sierra Leone for example, there is a lot of cross-border travel for both commerce and social reasons. Commerce-related travel is almost exclusive to men and therefore more likely to impact their MDA access.

While the qualitative data from this study shows little evidence of people with disabilities being systematically missed by MDAs, some challenges were cited in the literature. A similarly structured qualitative study [35] demonstrated that disability was cited as a barrier to MDA in Liberia and Nigeria NTD programs. Findings from this study showed the importance of engaging people with disabilities in MDA strategies, with some people stating that door-to-door distribution was the best method for reaching people with disabilities while others preferred to be reached with MDA at the places where they congregated to beg [35].

Additionally, people who are unable to stand due to a physical disability may not be measured properly (using a dose pole) for the appropriate dosing for ivermectin. During FGDs, CDDs reported using different strategies in these circumstances, but these should be standardized to ensure they are applied uniformly and accurately, and CDDs should demonstrate sensitivity in their application.

In Sierra Leone, MDAs are conducted differently in towns and cities than in villages, where nursing students or other non-community health workers (CHWs) conduct the drug distributions. These distributors are often not from the community and may lack relationships or established trust with community members. This sometimes appeared to impact their ability to reach all the eligible household members, especially men, according to the CDDs working in urban communities. One male respondent from an urban neighborhood in Kenema said, “*The CDDs are concentrating more in the homes and forgetting us in the business places*. *Like the office where I am working*, *they have never been there*, *and I do not have time to go and meet them*. *So*, *for us in the business places*, *access is very low*.”

### Social and behavior change (SBC) messaging and access to information about NTDs and MDAs

Among Act | West study participants, levels of sensitization and knowledge about both NTDs and the associated MDAs varied greatly between districts in Sierra Leone, Ghana, and Côte d’Ivoire as well as by sex of the respondent, with rural sites and those with higher prevalence of LF in particular having greater awareness. Both male and female study respondents reported that women have more access to information about the drugs and the diseases than men—except in Divo district, Côte d’Ivoire, where adult male community members appeared to be better informed about NTDs and transmission.

Across the three study countries, women have extensive networks with other women who share health information with each other. According to female community members in each of the three study countries, they are also more likely than men to hear the announcements made by health staff in the information centers, through info-vans, after the village gong sounds, and in churches and mosques—even though the messages are meant for everyone. Men generally have less interaction with health staff and are away from their homes and communities more often than women. During the day, they are mostly on their farms or places of work and lack access to concrete information about NTDs and MDAs.

In rural areas, typically information is shared about upcoming MDAs in public places by town criers who are trained by district health teams before each campaign and then inform community members about when, where, and why they will take place. However, women are more likely to hear messages from town criers because they are more likely to be in town doing trading, whereas men tend to be away from the village for farming or work.

Women are home or in the community much of the time and are therefore more likely to be reached with messages through multiple sources (e.g., from health workers and town criers). Men who are frequently absent from the community are more likely to miss these messages, particularly in urban areas. For instance, during FGDs in Kenema and Western Area Rural districts of Sierra Leone, some fathers claimed to have never heard about the MDAs.

While some community leaders and members correctly identified the sources of transmission for each NTD, most participants could not explain how people contracted specific NTDs. In districts with low transmission rates of LF and onchocerciasis in particular, there was little understanding of how people contracted the diseases, and few people knew anyone who was afflicted. Among community members, the only people who knew anything about NTDs were those who knew someone suffering from one, highlighting the challenge for the campaigns during the last mile of MDA where people no longer see the need for the drugs.

Some attributed the diseases to witchcraft. “*People don’t understand how diseases are transmitted*, *particularly NTDs*. *They think it is caused by sorcery*. *Need to destigmatize and demystify these diseases*, *particularly those that cause visible disability*.”–KII, Côte d’Ivoire. Such misconceptions around the cause of diseases could impact people’s risk perception and therefore acceptance of MDA.

Information about possible side effects of the drugs for children or adults is not always shared with community members. Sometimes children and their parents are unprepared for the dizziness, fatigue, skin itchiness, and vomiting that comes with the ingestion of the drugs, which some male focus group participants claimed made them hesitant to take the drugs in subsequent MDAs. This finding is supported by several other studies, which showed that experiences of adverse events among participants would sometimes affect participation in future MDAs [36]. Many community members across all three countries expressed a desire to have more information about the targeted NTDs, as well as potential side effects of the medications distributed. While both men and women requested more information and visual aids, only men cited lack of communication as a reason for refusing to take the drugs.

### Household power dynamics and decision-making for MDA participation

Most women across the three countries reported being able to make decisions about their own health care, including taking drugs through the MDAs. However, in Kpandai district of Ghana (a conservative district in the north), men indicated that women need consent from their husbands to take the drugs. Though, when women in the same communities were asked if decision-making structures in the family impeded them from ingesting the drugs without their husbands’ permission, nearly all said they were able to take the medicines even if their husbands were not home. Furthermore, they indicated that they received their husbands’ pills from the CDDs so that the men can ingest them when they come home later in the evening. This indicated that CDDs in this district did not always observe treatment directly, therefore it was not possible to reliably track ingestion of the drugs in this case.

### Acceptance of MDA

The majority of men, women, and children that participate in the MDAs do so based on preregistration systems, community sensitization, trust of the CDDs, trust in the government, and the perceived efficacy of the drugs, according to FGDs among community members, CDDs, and health providers, as well as the majority of KIIs. The MDAs have generally been successful, according to both program coverage data and qualitative responses from study participants.

Marginalized ethnic groups, such as the Fulani in some areas of West Africa, have been difficult to reach during MDAs due to both access issues and refusal to participate. Challenges include nomadic lifestyles, mistrust towards local populations, and strong patriarchal social norms that do not allow women to be engaged by male CDDs of a different ethnic group. *“Fulani are very conservative and are resistant*, *and women refuse to take the drugs if their husband is out and can’t give permission*. *Fula depend on their tribal leaders to tell them whether to take it*.”–KII, Sierra Leone. However in countries where engagement of cultural and religious leaders has been prioritized to improve trust and communication between the program and minority ethnic groups, such as in Sierra Leone where national Fulani leaders were engaged to conduct outreach to communities, uptake has improved among these groups.

In most countries, the government NTD programs attempt to match CDDs from the same ethno-linguistic group as the community where they are leading drug distributions. However, this has been a greater challenge in urban areas where CDD catchment areas often include households from multiple ethnic and linguistic groups. Given the importance of maintaining trust between community members and CDDs, great care should be taken to prioritize assigning CDDs who share a common language or ethnicity with the targeted community. This is particularly important in areas or communities where there may be historical mistrust—such as among Fulani communities in Sierra Leone, and in Ghana where mistrust among rival political parties, often falling along tribal lines, was mentioned during interviews and focus group discussions. This issue also surfaced in the literature through a study on MDA participation in Sudan, where some nomadic communities refused treatment from Sudanese health workers of a different ethnicity because of mistrust between the two ethnic groups [37].

According to our study, people generally believed that the drugs are effective; however, respondents had some misconceptions regarding how people contract the diseases and were subject to misinformation about the drugs, which impacted their perception of risk and therefore their willingness to participate in MDA. A few respondents in each country reported that some people rejected the drugs because they believed rumors about the MDAs, for instance, that the drugs are part of a secret family planning campaign. This mistrust of government-issued drugs was reinforced by misinformation circulating on social media. “There is a lot to prejudge: it is to make the population sick, it is to make men sexually inactive or the women sterile, it is to stop the birth rate in Africa.”–Provider, FGD, Korhogo district, Côte d’Ivoire.

Additionally, CDDs reported that it was sometimes hard to convince people with disabilities to participate in MDA. This was especially true of individuals with lymphedema, hydrocele, or onchocerciasis-related blindness. Many times, these individuals refused to take the drugs because they thought it was too late for them to benefit.

Male and female community members as well as CDDs reported that men were more likely to refuse to take the drugs. This tracks with findings from other research demonstrating that in some cases men were often more suspicious of MDA and less likely to comply with treatment [15]. This mistrust among men can also impact access to treatment for their wives and children if they refuse to let them participate, according to focus group discussions in both Ghana and Sierra Leone. In this case, the true gender dimensions around access and participation may be hidden within the data since it is not just men who are left out [35]. An anecdote was recounted during the qualitative work in Côte d’Ivoire regarding a town crier in Korhogo district who promoted the MDAs to community members but then (subversively) told male friends not to take the drugs. However, this appeared to be an isolated incident.

In Sierra Leone, MDAs are part of a long-standing and well-established countrywide program. Some of the CDDs in Sierra Leone who participated in the FGDs, particularly in Koinadugu district, had supported the MDAs for over a decade. Most community participants, especially in rural areas, expressed gratitude for the drugs. CDDs, MDA supervisors, and Ministry staff said they themselves had experienced positive health effects and seen improved health across their communities. Many community members as well as CDDs and NTD program officials mentioned that when MDAs first began more than a decade ago, there was a great deal of mistrust regarding the drugs, and high levels of negative side effects were also reported. Male participants spoke about negative side effects more often than women, although most reported that they only experienced negative side effects the first time they took the drugs, making them hesitant to take the drugs again, but most had taken them in following years with no problems. Only a handful of male participants in Sierra Leone admitted to refusing to participate in MDA. They said they refused because they mistrusted the drugs or feared side effects and did not understand the need to take medication if they did not feel ill.

On the other hand, some men across the three study countries reported the drugs improved their sexual performance or stated they felt stronger or more vital after taking the drugs, providing them with a positive incentive to participate.

A few CDDs in Sierra Leone stated that some girls and adolescent boys have resisted because they feared the drugs would make them sterile, indicating a need for increased messaging on NTDs and the drug campaigns. “*Women who aren’t sure they are pregnant may not take it*. *Some girls may think it is meant to be a contraceptive*, *so they might be hesitant to take it*.*”*–KII, Sierra Leone

As mentioned above, individuals who experienced negative side effects from any of the MDA drugs were often less willing to participate in a subsequent MDA. CDDs cited many difficulties convincing people to take the drugs if they experienced particularly bad side effects, which is understandable; however, improving communication around potential side effects before MDA could ease concerns. During FGDs, some men who had refused to participate in MDA in the past reported that they would be more likely to take the drugs if the CDDs took more time to explain the potential side effects, so they knew what to expect. Community members did not appear to know that itchiness is an indicator of the drug’s (albendazole) effectiveness in killing STH in the body. Information linking side effects with the level of efficacy is likely to improve people’s acceptance. “*Some men who were highly paracitized had high side effects*, *so they refuse in the future*.”–KII, Sierra Leone

Another aspect to consider when thinking about equity in MDA coverage is that distributors are supposed to directly observe treatment to ensure completion. In some countries, however, treatment is provided to households, but ingestion is not directly observed. In Ghana, many CDDs reported doing their best to administer the drugs to everyone, despite many challenges. Despite explicit guidance to watch everyone ingest the drugs, CDDs did not always practice this in all communities. Some CDDs reported and community members verified that CDDs would leave the drugs with women of the household if not everyone was home at the time of the visit. However, women admitted they could not always verify if men took the drugs. Also, prior research found that gender-related power dynamics can increase the risk that women who receive drugs without directly observed treatment by CDDs may not in fact ingest the drugs due to a variety of factors, including lack of knowledge on the benefits of MDA, low perception of risk, mistrust, or medications being given to another family member [6].

### Selection of CDDs and their perceived effectiveness by gender

According to data available from USAID-supported NTD projects in 16 countries between 2012−2017 [38], MDA training rates for three categories of staff, CDDs, other MDA staff, and trainers/supervisors, were almost universally higher for men than women. While useful, the study by Shoemaker et al. [38] only looked at the breakdown of CDDs trained (as this was the only data available) and did not consider sex-disaggregated data on CDDs actually conducting mass drug administration, nor did it consider attrition rates or experiences and effectiveness of the CDDs. For example, at the national level, numbers of CDDs trained in 2014, 2015, and 2017 (our proxy for CDDs employed) showed a higher number of female CDDs than males in Ghana [38]. However, district health officials and key informants stated that this was not the case in the three districts visited for this study. Therefore, we can determine that program countries are generally training and engaging women at proportions far below equitable levels.

The vast majority of CDDs in Sierra Leone at the time of this study (2019) were male. The reasons given were that women are busy with farming and household responsibilities; their schooling hasn’t been prioritized (disadvantaging them for CDD work and other jobs where literacy is required); and some husbands are jealous or disapprove, particularly when there is a need to travel outside the immediate community or work late hours to complete the distributions. Several CDDs also stated that the small incentive payments (or lack of incentives) are a greater barrier for women, due to their numerous other responsibilities.

In Côte d’Ivoire, low literacy among women in rural areas was cited as one of the primary reasons there are fewer females than males as CDDs. Both men and women reported that women do not have time to volunteer as CDDs because of their household chores or their husbands would disapprove. A few expressed concerns about the safety of women traveling alone in rural areas—especially because they often walk, whereas men more often have access to motorbikes to travel between communities. “Men do not accept that their women participate in this kind of activity; women are too busy with farming activities; the illiteracy rate is too high compared to men.”–KII, Touba district, Côte d’Ivoire

Globally, in some societies, it is considered inappropriate for women to travel outside the home or to travel from house to house, making this line of work challenging for women to take on. In one study from Kenya, some female CDDs reported physical or emotional abuse from their husbands for coming home late after distributing drugs, and some female CDDs had to justify their work as CDDs to their husbands [39].

Evidence exists from the global literature suggesting that MDA programs delivered by female CDDs can achieve equal or greater coverage with less participant attrition compared to male counterparts [40–43]. Community members often reported female CDDs as more committed, persuasive, and patient than men [40], and some studies have identified underutilization of female CDDs as one reason for limited effectiveness of ivermectin distribution in the treatment of onchocerciasis [40]. One study in Uganda found that female CDDs outperformed male CDDs [44]. According to the end of program report for *End NTDs in Africa*, recruiting women as CDDs was an effective strategy in Niger, increasing coverage in many areas, because women can enter households whereas men are not always allowed to do so [45]. However, another study in Uganda found that social hierarchies impacted effectiveness of female CDDs, as younger women were not able to insist that older men take the pills in their presence [44,46].

Among participants in the Act | West study, feedback on the effectiveness of female CDDs was overwhelmingly positive, according to community members, community leaders, and NGO and ministry staff. Importantly, female CDDs often have more access to other women in very conservative homes, where men are not allowed to talk or mix with women. Women can confide more easily in other women on issues considered private to them, such as pregnancy.

Many respondents (both men and women) felt that female CDDs express themselves much better than men; are more reliable and patient; and take more time to explain to men and women what the diseases are, how they affect people, why people should take the medicine, the side effects of the drugs, and what people should do if they experience side effects. They said female CDDs typically meet their targets and have impressive record-keeping abilities. Female CDDs mentioned that male CDDs do not always know how to communicate effectively to people about the diseases and reasons people should take the drugs, which can be a barrier to access and treatment. “*Women are more convincing than men to encourage people to take the drugs*. *Women want to protect children*.” -KII, Côte d’Ivoire.

Despite the ringing endorsement by community members and MDA supervisors (across the districts) of female CDDs’ abilities, community leaders reported a clear preference for choosing male CDDs. When asked why people thought there were more male CDDs in the communities, both men and women said they thought men can do a better job than females traveling to hard-to-reach and insecure areas and that women are “weaker” than men and should stay home to take care of the children. This finding aligns with research from the 2016–2019 WHO project, *Integrating a Gender*, *Equity*, *and Human Rights Focus into National Programming on Preventative Chemotherapy and Transmission Control for NTDs*, which found that the selection of CDDs was mostly determined by men, who reported not selecting women due to an opinion that women were too weak to take on the role [47].

### Gender-related benefits and challenges to performing the role of CDD

CDDs are often well respected by the communities they serve and have a say in community issues. They are seen as role models and are viewed as providing a vital service to the community. For some men, being a CDD is a pathway to becoming a revered community leader. In the Act | West study sample of female CDDs, when asked if their husbands were supportive of their work as CDDs, all said yes. Some of their husbands are also CDDs so they work together on the distribution. In Sierra Leone, both male and female CDDs felt that the main benefits of serving in this role are being respected by the community and feeling good about helping their neighbors and friends. Both groups reported that being CDDs has elevated their social status. Many were selected to become paid community health workers (CHWs) due to their CDD role when Sierra Leone rolled out a new CHW program. When female CDDs were asked why they perform this work even though it is physically demanding, can be stressful, and does not pay any salary, they said they feel satisfaction in helping fellow community members stay healthy.

The literature review supported these findings that the increased responsibility of the role of CDD can lead to greater recognition and support in terms of both career and social advancement, which is particularly helpful for female CDDs [37]. In other studies, female health volunteers reported this role as empowering and leading to increased mobility, knowledge [48], feelings of accomplishment, and respect from husbands and family members [36].

However, both men and women CDDs experienced challenges in the role, including traveling long distances by foot or bike to deliver the drugs, often in heavy rain; insufficient supply of drugs (particularly in urban and border communities); overwork; too little notice to access the schools for distributions; difficulty accessing remote and mountainous communities (particularly in the rainy season); skepticism towards CDDs by community members; physical barriers such as gated compounds (especially in urban areas); and difficulty with acceptance within communities of certain ethnic minority groups. The long distances and long hours were seen as a larger barrier for women than men, since men often have access to motorbikes, bicycles, and fewer household responsibilities to work around.

Of the 11 countries Act | West is working in, only Sierra Leone and Togo pay their CDDs. In the context of the larger health system, paid positions in the health sector are often held predominantly by men. From the information available in the final project report from *End in Africa*, women were trained for paid positions at much lower rates than men, meaning that while women may be given opportunities to participate in the volunteer cadres of health systems in many sub-Saharan African countries, they are often excluded from the more respected and financially beneficial roles.

In the three countries where this study was conducted, both the Ministry officials we spoke to and the CDDs themselves confirmed that CHWs (often the same people) doing other types of health outreach and community work received higher stipends for those activities than for the MDAs. This created resentment and discouragement, given that the MDA work is often more intense and physically demanding (given the door-to-door distribution) than other health campaign work. In circumstances when other health programs within the same countries are providing higher incentives, it causes MDA work to be less appealing to potential CDDs.

While relying on unpaid volunteers to implement MDAs can be a challenge across the board, this can be particularly harmful for female CDDs, unintentionally reinforcing norms that encourage women to participate in uncompensated labor [49]. Additionally, since women and girls most often do the majority of housework and caretaking, both unpaid labor and the devaluing of female CDDs’ time reinforces gender stereotypes that caretaking is a women’s role and does not merit financial remuneration [49]. This was confirmed in the Act | West study, where female CDDs cited it as a much greater barrier than male CDDs.

Within the Act | West study, female CDDs who traveled together in pairs sometimes mentioned safety issues during focus group discussions; female CDDs paired with male CDDs were less likely to cite such concerns. Female CDDs complained that they are sometimes harassed by community men if they are working in a remote area, and they said they did not feel comfortable traveling without a male CDD during the evening hours.

Ensuring that women are selected, trained, and supported to act as CDDs as part of MDA programs has the potential to elevate the social status of women and provide them rewarding and valuable skills and experience [50], as well as improve program reach. However, given that in most contexts, women are responsible for the vast majority of domestic duties, the additional responsibility of drug distribution may overburden female CDDs [39,50] if not financially compensated.

## Discussion

NTD programs can promote gender equity through increasing women’s participation in human resources for the program whether as CDDs or in leadership positions. Increased involvement by women as CDDs is critical since it may increase the proportion of females reached as well as improve MDA outcomes in general. This can be accomplished through sensitizations on gender dynamics and the value of more women serving as CDDs for NTD program officials from the MOH as well as districts and community members.

Although women CDDs often reported non-monetary benefits of the CDD role, including increased social standing and respect in their community and families, financial compensation should be the long-term goal for all CDDs. Financial compensation for CDDs can improve gender equity by reducing the unpaid work burden that women in general, and female CDDs specifically, face and encourage more women to volunteer for the role, as well as improving retention and motivation of trained CDDs of all genders.

In addition to compensation and sensitization, it is essential to collect and use more accurate data on the gender make-up of the CDD workforce. Almost all available data for CDD sex is based on training data, which is a proxy but doesn’t provide the full picture. We recommend all NTD programs add a space on MDA data collection forms to track and report CDD sex within monitoring systems all the way to the national level and for this data to be reviewed by district and national teams annually to identify gaps and communities falling behind on the CDD gender parity.

NTD programs should prioritize improving inclusive messaging and information about NTDs and MDA such as providing CDDs with gender sensitive and disability inclusive visual aids and messages adapted for low literacy populations; increasing mass media messaging in urban areas or other areas with low coverage; and supplementing CDD training curricula with modules that provide more information about the NTDs themselves, modes of transmission, importance of direct observation for ingestion of the drugs by CDDs, potential side effects of the drugs for children and adults, drug safety profiles for pregnant and breastfeeding women, and ways to dispel myths about the drugs. Since men have less interaction with health providers and are away from their homes and communities more often than women, they need to be reached through tailored approaches such as through radio or during evenings or early mornings when they are more likely to be home.

Another approach could be to pilot a cadre of mostly female volunteer “MDA Champions” to undertake SBC and outreach on NTDs and MDAs in areas where refusal rates are high or in disease hotspots. These volunteers would not engage in drug distributions; rather they could prime the communities for the MDAs a few days in advance to increase awareness, help alleviate concerns about side effects, dispel myths about NTDs and MDAs, and reduce stigma towards people with NTDs.

To ensure access and acceptability of MDA for people with disabilities, programs should provide job aids to CDDs that include how to do dosing of Ivermectin for people who aren’t able to be measured using a dose pole, and how to implement disability inclusion and equity through MDA delivery strategies within broader CDD training.

Challenges in program coverage in communities close to an international border can impact both men and women but are likely to result in more men being missed for reasons discussed in the results section. It is essential therefore to improve coordination across neighboring countries to increase coverage in border areas. This can be accomplished by scheduling MDAs at the same times for both sides of border districts so that travelers and migrants are reached even if they are away from home. This can be a challenge in the case of neighboring countries with different NTD program funding sources and drug procurement schedules, but planning meetings between neighboring NTD programs could improve communication and coordination in planning MDAs.

NTD program teams should continue to engage with leaders of Fulani and other marginalized ethnic communities to sensitize them to the availability of the drugs and discuss more effective and culturally-sensitive ways CDDs can conduct MDA within those minority communities. Ensuring that CDDs are from the same ethnic group as community members is a greater challenge in urban areas where CDD catchment areas often include households from different ethnic and linguistic groups. Given the importance of maintaining trust between community members and CDDs, great care should be taken to prioritize assigning CDDs who share a common language or ethnicity with the targeted community. This is particularly important in areas or communities where there is historical mistrust—such as among Fulani communities in Sierra Leone, and in Ghana where mistrust among rival political parties, often falling along tribal lines, was highlighted as a challenge for MDA programming.

Since 2021, the Act | West program has supported Ministries of Health to begin implementing many of these recommendations across Ghana, Côte d’Ivoire, and Sierra Leone. It will be critical to track impacts of efforts to improve equity and inclusion across NTD programs through the review of sex-disaggregated monitoring data on CDD sex, program coverage and subsequent program reviews, TAS and pre-TAS failure investigations, or other studies using a gender and social inclusion lens to rapidly identify gaps and needs at all levels of NTD programming. Addressing gender-related dynamics and improving social inclusion of minorities and people with disabilities as well as nomadic and highly mobile populations will not only improve equity in NTD programs but will also improve overall elimination and control of NTDs targeted by MDAs.

### Study limitations

Given the nature of the study as qualitative research, study participants were not randomly sampled out of the population, therefore raising the possibility for selection bias in community leaders and health care workers selecting “model” respondents. Additionally, despite efforts to reduce acceptability bias in answering questions through tool design and researcher training, it is always a possibility that some participants answered how they assumed the researcher wanted them to rather than what they truly believed or felt.

Another limitation was the perceived lack of direct participation by persons with known disabilities in the FGDs or KIIs. Our objective was to include one or two people with disabilities in each community FGD, but this proved to be more challenging than anticipated. The team reached out to disabled persons organizations (DPOs) in each country, conducted interviews with representatives from those DPOs, and asked questions about disability and inclusion in each FGD and KII. But no people with disabilities were available or met the inclusion criteria for participation in any of the communities—with the exception of one community in Côte d’Ivoire. We therefore based our findings of disability inclusion on the results of interviews with CDDs, health officials, and non-disabled community members.
